# Pulmonary Blastomycosis in Two Immunocompetent Patients: The Role of Obesity and Vitamin D Deficiency

**DOI:** 10.7759/cureus.70366

**Published:** 2024-09-28

**Authors:** Strahinja Gligorevic, Nebojsa Brezic, Andrew Petcu, Erik Sviggum, Igor Dumic

**Affiliations:** 1 Internal Medicine, University of Belgrade School of Medicine, Belgrade, SRB; 2 Anesthesiology, Resuscitation, and Critical Care, University Clinical Center of Serbia, Belgrade, SRB; 3 Hospital Medicine, Mayo Clinic Health System, Eau Claire, USA; 4 Radiology, Mayo Clinic Alix School of Medicine, Rochester, USA

**Keywords:** blastomycosis, community-acquired pneumonia, immune dysfunction, obesity, vitamin d deficiency

## Abstract

This case report depicts two patients with morbid obesity who presented to the ED with signs and symptoms of community-acquired pneumonia and were treated accordingly. Despite empiric antibiotic therapy, their symptoms did not subside, prompting further evaluation, which revealed pulmonary blastomycosis. Both patients were also found to have severe vitamin D deficiency. Treatment with amphotericin B followed by itraconazole, along with aggressive vitamin D supplementation, led to clinical improvement and resolution of lung lesions in both cases. Although blastomycosis is not rare in immunocompetent individuals, its severe forms are usually associated with underlying immunosuppression or significantly high inoculum. Blastomycosis presents a diagnostic challenge due to its nonspecific symptoms and radiographic findings. This case series underscores the importance of considering blastomycosis in the differential diagnosis of persistent pneumonia in obese individuals, particularly in endemic areas. It also suggests that vitamin D deficiency may play a role in disease susceptibility and severity. This report contributes to existing medical literature by emphasizing the potential link between obesity, vitamin D deficiency, and the risk of blastomycosis, highlighting the need for further research into this association.

## Introduction

Blastomycosis, an uncommon fungal infection in non-endemic areas, is primarily acquired through the inhalation of conidia of one of the two thermally dimorphic fungi: *Blastomyces dermatitidis* or the more recently described *Blastomyces gilchristii* [1]. The newly emerged *Blastomyces helicus* has been found to cause illness in the western United States but remains poorly understood [2]. These fungi are endemic in the soils of the Ohio and Mississippi River Valleys, the St. Lawrence River basins, the Great Lakes region, and the southeastern United States [3]. In 2019, the Centers for Disease Control and Prevention (CDC) received 240 case reports of blastomycosis from five states where reporting new cases is mandated (Arkansas, Louisiana, Michigan, Minnesota, and Wisconsin). The collective incidence of blastomycosis in these states stood at 0.8 cases per 100,000 population, with Minnesota and Wisconsin accounting for 75% of all reported cases [4].

Clinical presentation of blastomycosis is very heterogenous and can vary from asymptomatic infection to acute respiratory distress syndrome [5,6] with multiorgan involvement [7]. Pulmonary infection is the predominant form of blastomycosis, with spores entering the body through inhalation. Acute pulmonary blastomycosis may resemble bacterial community-acquired pneumonia (CAP), presenting as fever, chills, cough, dyspnea, chest pain, and malaise [8]. Chest imaging is nonspecific and typically reveals consolidation as the most common presentation [9]. Due to the nonspecific nature of symptoms and a low index of suspicion, diagnosis is frequently delayed [7]. *Blastomyces *has the potential to disseminate, impacting multiple organs in 25-40% of symptomatic cases. Extrapulmonary involvement most commonly affects the skin, bones, and central nervous system [7], though bizarre manifestations, such as primary tracheal tumors, have also been documented [10].

Obesity is a growing concern in the United States, with the CDC reporting that 42.4% of the adult population is obese [11]. Obesity is a known risk factor for cardiovascular diseases, diabetes, certain cancers, metabolic dysfunction-associated steatotic liver disease (MASLD), chronic kidney disease, sleep apnea, and depression [12]. Obesity also increases susceptibility to both CAP [13] and healthcare-associated pneumonia [14,15]. Despite its traditional association with protracted low-grade inflammation, recent research has highlighted obesity's significant detrimental impact on immunity [16]. Furthermore, obesity is strongly associated with vitamin D deficiency, with obese individuals having a 35% higher prevalence of deficiency compared to those with a normal weight and 33% of obese adults being deficient in vitamin D [17]. Given that vitamin D plays a crucial role in modulating both adaptive and innate immune responses [18], its deficiency in obese individuals further compromises immune function [19].

## Case presentation

Case 1

A 38-year-old White man from Wisconsin presented to the ED with complaints of fever, worsening shortness of breath, productive cough, and left-sided chest pain that had progressively worsened over the preceding two weeks. Two days after the onset of symptoms (12 days before the current admission), he was prescribed amoxicillin/clavulanic acid tablets (875 mg/125 mg) twice daily and doxycycline capsules (100 mg) twice daily for presumed CAP. Despite adhering to the prescribed medication regimen for one week, his health continued to deteriorate. Apart from class III obesity, the patient had no other chronic medical conditions and was not using any medications or over-the-counter supplements. He reported a negative history of smoking, tobacco chewing, alcohol consumption, or illicit drug use. He was in a monogamous heterosexual relationship and denied any risk for sexually transmitted infections. He worked at a factory producing air filters and denied exposure to soil dust or pet contact. Physical examination revealed a morbidly obese man with a BMI of 45 kg/m^2^. On presentation, he was hypoxic with an oxygen saturation of 86% on room air, necessitating 4 L/min of nasal cannula flow to maintain oxygen saturation above 92%. He exhibited mild-to-moderate distress due to tachypnea of 30 breaths per minute but had normal blood pressure and heart rate. His temperature was 38.1 °C. Auscultation revealed regular heart sounds with distant heart sounds and significant rhonchi in the left lower lobe. There was no lower extremity edema, rashes, or joint swelling. Upon admission, the patient received IV levofloxacin 750 mg daily (obesity dosing), and a comprehensive workup was initiated.

**Figure 1:**
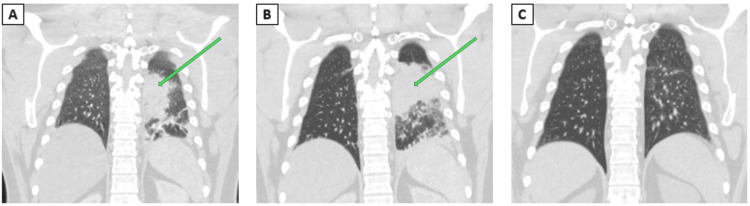
Chest CT of Case 1 (A) Left lower lobe airspace opacification on initial presentation marked by a green arrow. (B) Worsening left lower lobe opacification five days after initial presentation marked by a green arrow. (C) Resolution four months after the initial presentation CT: computed tomography

CT of the chest with IV contrast revealed consolidation in the left lower lobe without mediastinal lymphadenopathy, as seen in Figure 1. Comprehensive workup for CAP that failed appropriate outpatient treatment is illustrated in Table 1. Given the absence of typical risk factors for fungal pneumonia in this patient, a vitamin D level was obtained, revealing severe deficiency at 3 ng/mL (normal values above 40 ng/mL).

**Table 1:** Illustration of extensive workup done as a diagnostic protocol for Case 1 PCR: polymerase chain reaction, HIV: human immunodeficiency virus, SARS: severe acute respiratory syndrome

Test	Blood specimen	Urine specimen	Sputum specimen	Respiratory nasopharyngeal swab
Cultures	Negative	Negative	Negative	-
Gram stain	-	-	Negative	-
Procalcitonin	Normal	-	-	-
1,3-β-D-glucan (BDG)	Negative	-	-	-
Galactomannan	Negative	-	-	-
Fungal smear	-	-	Positive for *Blastomyces dermatitidis/gilchristii*	-
Fungal culture	-	-	Positive for *Blastomyces dermatitidis*	-
Histoplasma antibodies	Negative	-	-	-
Histoplasma antigen	Negative	Negative	-	-
Histoplasma PCR	-	-	Negative	-
*Aspergillus* antigen	Negative	-	-	-
*Blastomyces* antibodies	Negative	-	-	-
*Blastomyces* antigen	Positive	Positive	-	-
*Blastomyces* PCR	-	-	Positive	-
*Coccidioides* antibodies	Negative	-	-	-
*Pneumocystis jirovecii* PCR	-	-	Negative	-
*Legionella pneumophila* antigen	-	Negative	-	-
*Streptococcus pneumoniae* antigen	-	Negative	-	-
Quantiferon TB-gold	Negative	-	-	-
*Mycoplasma pneumoniae* antibodies	IgM and IgG negative	-	-	-
*Chlamydia pneumoniae* antibodies	IgM negative	-	-	-
*Chlamydia trachomatis* antibodies	IgM and IgG negative	-	-	-
*Chlamydia psittaci* antibodies	IgM and IgG negative	-	-	-
HIV-1/-2 antigen	Negative	-	-	-
Adenovirus	-	-	-	Negative
Coronavirus 229E	-	-	-	Negative
Coronavirus HKU1	-	-	-	Negative
Coronavirus NL63	-	-	-	Negative
Coronavirus OC43	-	-	-	Negative
SARS coronavirus-2	-	-	-	Negative
Human metapneumovirus	-	-	-	Negative
Human rhinovirus/enterovirus	-	-	-	Negative
Influenza A virus	-	-	-	Negative
Influenza B virus	-	-	-	Negative
Parainfluenza virus 1	-	-	-	Negative
Parainfluenza virus 2	-	-	-	Negative
Parainfluenza virus 3	-	-	-	Negative
Parainfluenza virus 4	-	-	-	Negative
Respiratory syncytial virus	-	-	-	Negative
Bordetella parapertussis	-	-	-	Negative
Bordetella pertussis	-	-	-	Negative
Chlamydia pneumoniae	-	-	-	Negative
Mycoplasma pneumoniae	-	-	-	Negative
Borrelia burgdorferi	Negative	-	-	-
Anaplasma phagocytophilum	Negative	-	-	-
*Borrelia miyamotoi*, PCR, B	Negative	-	-	-
Ehrlichia chaffeensis	Negative	-	-	-
Ehrlichia ewingii/canis	Negative	-	-	-
Ehrlichia muris eauclairensis	Negative	-	-	-
*Babesia divergens*/MO-1	Negative	-	-	-
Babesia duncani	Negative	-	-	-

After testing demonstrated infection with *Blastomyces spp.* amphotericin B was started. The patient received 5 mg/kg IV amphotericin B daily for 10 days, which he tolerated well aside from experiencing mild infusion-related chills and shivering, with no other reported side effects. Furthermore, the patient received aggressive vitamin D supplementation, consisting of 50,000 international units (IU) weekly for eight weeks, followed by 5,000 IU daily thereafter. Clinically, he demonstrated improvement and was subsequently transitioned to oral itraconazole at a dose of 200 mg twice daily. Before initiating itraconazole, an electrocardiogram was obtained, revealing a normal QT segment. One week later, itraconazole levels were assessed and found to be within therapeutic range at 1.5 mcg/mL (goal above 1 mcg/mL), and thus the patient was continued on the same regimen.

Itraconazole levels, along with a complete blood count and comprehensive metabolic panel, were monitored at intervals of one to two weeks, and after six months of treatment, complete resolution of CT lesions was observed, and the patient returned to his baseline health status.

Case 2

A 42-year-old White man from Wisconsin presented to the ED in early February with persistent cough, shortness of breath, chest pain, and intermittent fevers since mid-December. His medical history included obesity (BMI 40.39 kg/m²), gout (not currently treated), bilateral carpal tunnel release, and hernia repair. He was not on any regular medications and had no significant smoking or alcohol history. He worked as a restaurant cook and had no recent travel or known tuberculosis exposure but had a previously healthy pet dog. Initially, he experienced fever, nasal congestion, and cough, which resolved after two weeks of symptomatic treatment. However, he returned to the ED in mid-January with sudden right-sided pleuritic chest pain and a productive cough. He was diagnosed with right upper lobe pneumonia and prescribed a five-day course of amoxicillin/clavulanic acid tablets (875/125 mg) two times per day and doxycycline (100 mg) two times per day for five days. His symptoms briefly improved but recurred post-antibiotics, prompting another ED visit where his symptoms were attributed to a viral illness or recently treated pneumonia.

A week later, he returned to the ED with coughing spells, bilateral chest pain, and intermittent fevers. A chest X-ray showed an increased right mid-lung infiltrate. He was admitted and treated with IV levofloxacin (750 mg daily) for one day, ceftriaxone (1 g every 24 hours), and doxycycline (100 mg every 12 hours) for two days, improving before discharge on cefdinir (300 mg) two times per day and doxycycline (100 mg) two times per day. However, in mid-February, he returned with a worsening cough, shortness of breath, sweating, and chest pain.

**Figure 2:**
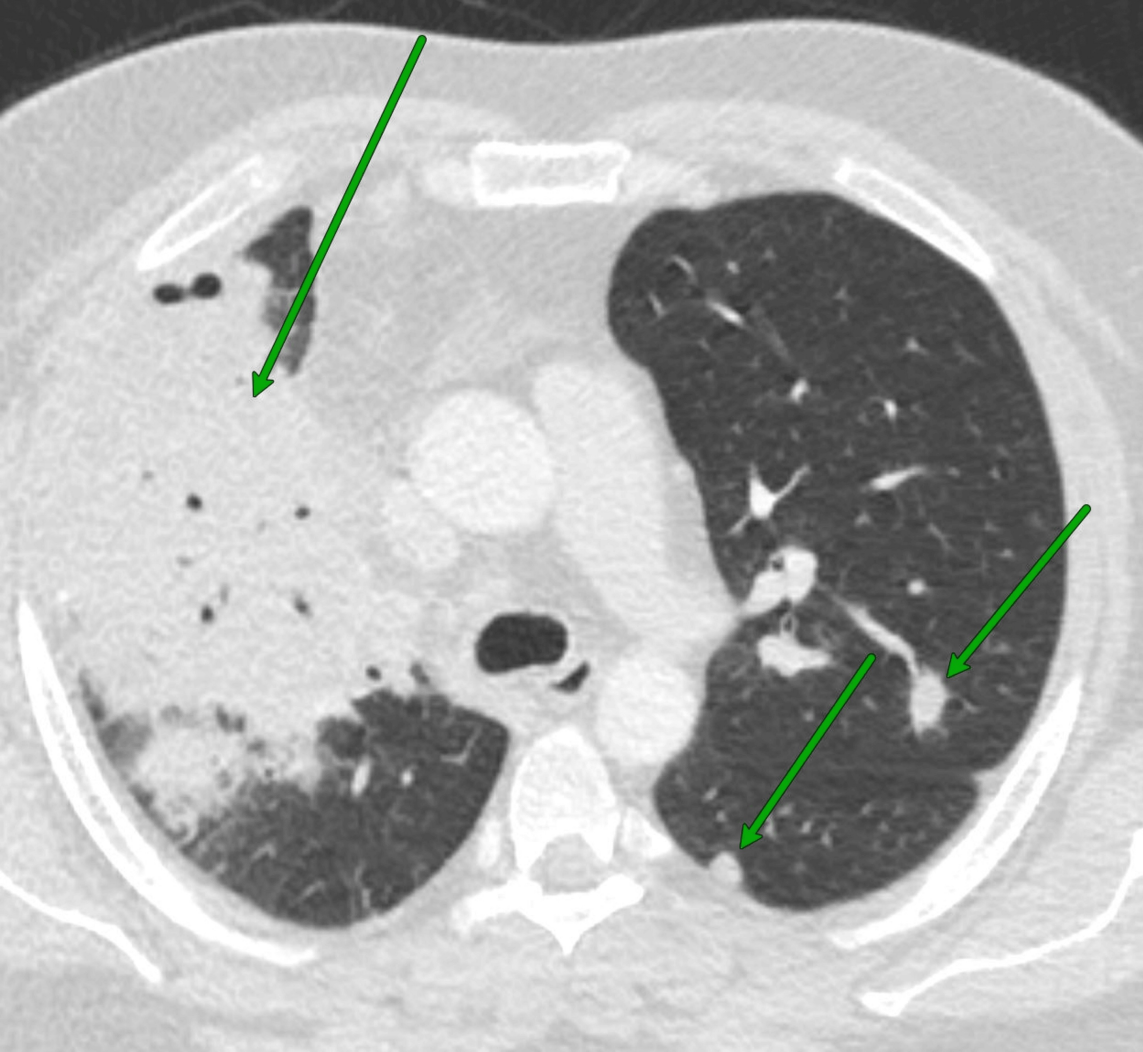
Chest CT of Case 2 Dominant right upper lobe consolidation, as well as infectious-appearing nodules in both the left upper and lower lobes marked by green arrows CT: computed tomography

The physical exam showed tachycardia and tachypnea but normal oxygen saturation. Blood work indicated worsening leukocytosis (20.8 x 10⁹/L compared to 17.1 x 109/L one week prior) and CRP (203.4 mg/L compared to 175.0 mg/L), D-dimer (3616 ng/mL compared to 556 ng/mL), and elevated procalcitonin (0.30 ng/mL). A CT chest angiogram revealed multifocal pneumonia, severe right upper lobe pneumonia, possible pneumatoceles, bilateral pulmonary nodules, and lymphadenopathy, as seen in Figure 2. He was started on piperacillin-tazobactam (3.375 g IV every six hours) and vancomycin (1,500 mg IV every 12 hours) and then switched to cefepime, vancomycin, metronidazole, and azithromycin due to concerns of a fungal infection.

The antibiotics were changed on the second day to IV ampicillin-sulbactam (3 g every four hours, dose adjusted for patient’s weight and severity of illness) for the presumed associated lung abscess. Initial tests were inconclusive, but *Blastomyces *antigen tests returned positive, and fungal sputum culture grew filamentous fungi (extensive workup is presented in Table 2). On the third day, he was diagnosed with pulmonary blastomycosis and started on IV amphotericin B (5 mg/kg every 24 hours) and oral itraconazole (200 mg three times per day for two days loading dose followed by 200 mg twice per day, with target itraconazole above 1 mcg/mL). Continued treatment included IV ampicillin-sulbactam for presumed bacterial lung abscess. His symptoms improved after four days, and a repeat CT after two weeks showed reduced consolidations.

**Table 2:** Illustration of extensive workup done as a diagnostic protocol for Case 2 PCR: polymerase chain reaction, HIV: human immunodeficiency virus, SARS: severe acute respiratory syndrome

Test	Blood specimen	Urine specimen	Sputum specimen	Respiratory nasopharyngeal swab
Cultures	Negative	Negative	Negative	-
Gram stain	-	-	Negative	-
Procalcitonin	Normal	-	-	-
1,3-β-D-glucan (BDG)	Negative	-	-	-
Galactomannan	Negative	-	-	-
Fungal smear	-	-	Positive for *Blastomyces dermatitidis/gilchristii*	-
Fungal culture	-	-	Positive for *Blastomyces dermatitidis*	-
Histoplasma antibodies	Negative	-	-	-
Histoplasma antigen	Negative	Negative	-	-
Histoplasma PCR	-	-	Negative	-
*Aspergillus* antigen	Negative	-	-	-
*Blastomyces* antibodies	Negative	-	-	-
*Blastomyces* antigen	Positive	Negative	-	-
*Blastomyces* PCR	-	-	Positive	-
*Coccidioides* antibodies	Negative	-	-	-
*Pneumocystis jirovecii* PCR	-	-	Negative	-
*Legionella pneumophila* antigen	-	Negative	-	-
*Streptococcus pneumoniae* antigen	-	Negative	-	-
Quantiferon TB-gold	Negative	-	-	-
*Mycoplasma pneumoniae* antibodies	IgM and IgG negative	-	-	-
*Chlamydia pneumoniae* antibodies	IgM negative	-	-	-
*Chlamydia trachomatis* antibodies	IgM and IgG negative	-	-	-
*Chlamydia psittaci* antibodies	IgM and IgG negative	-	-	-
HIV-1/-2 antigen	Negative	-	-	-
Adenovirus	-	-	-	Negative
Coronavirus 229E	-	-	-	Negative
Coronavirus HKU1	-	-	-	Negative
Coronavirus NL63	-	-	-	Negative
Coronavirus OC43	-	-	-	Negative
SARS coronavirus-2	-	-	-	Negative
Human metapneumovirus	-	-	-	Negative
Human rhinovirus/enterovirus	-	-	-	Negative
Influenza A virus	-	-	-	Negative
Influenza B virus	-	-	-	Negative
Parainfluenza virus 1	-	-	-	Negative
Parainfluenza virus 2	-	-	-	Negative
Parainfluenza virus 3	-	-	-	Negative
Parainfluenza virus 4	-	-	-	Negative
Respiratory syncytial virus	-	-	-	Negative
Bordetella parapertussis	-	-	-	Negative
Bordetella pertussis	-	-	-	Negative
Chlamydia pneumoniae	-	-	-	Negative
Mycoplasma pneumoniae	-	-	-	Negative
Borrelia burgdorferi	Negative	-	-	-
Anaplasma phagocytophilum	Negative	-	-	-
*Borrelia miyamotoi*, PCR, B	Negative	-	-	-
Ehrlichia chaffeensis	Negative	-	-	-
Ehrlichia ewingii/canis	Negative	-	-	-
Ehrlichia muris eauclairensis	Negative	-	-	-
*Babesia divergens*/MO-1	Negative	-	-	-
Babesia duncani	Negative	-	-	-

He completed 15 days of IV amphotericin B and 14 days of IV ampicillin-sulbactam, continuing itraconazole post-discharge. At a three-month follow-up, he reported fatigue but otherwise tolerated treatment well, with therapeutic itraconazole levels. He was prescribed an additional three months of itraconazole to complete a total of six months of treatment. Notably, he was found to have a significant vitamin D deficiency (12 ng/mL, reference range 20-80 ng/mL) and was started on vitamin D3 supplementation (4,000 units by mouth daily for seven days followed by 2,000 units daily).

## Discussion

When inhaled, *Blastomyces *conidia induce granulomatous lung inflammation, which is usually cleared by the immune system before transitioning into yeast. However, in some cases, conidia evade defenses and develop into yeast, causing infections that can potentially spread from the lungs to the skin, bone, and the central nervous system [7]. Solid organ or bone marrow transplantation recipients, patients receiving tumor necrosis factor (TNF) inhibitors, and those with malignancy or AIDS are at higher risk of severe disease [7].

Well-established independent risk factors for severe disease include neutrophilia and lymphopenia at presentation. Both factors, along with advancing age, are identified as independent risk factors for increased mortality [3]. Lymphopenia upon presentation serves as a prognostic marker, reflecting the importance of cell-mediated immunity against the fungus. However, none of our two patients were lymphopenic. Conflicting evidence persists regarding the relationship between comorbidities, pharmacological immunosuppression, and the risk of severe disease and mortality. While certain studies identify pulmonary multilobar disease, obesity, diabetes mellitus, and immunosuppression as significant independent risk factors for severe blastomycosis and increased mortality [20-22], others do not find this association [3]. Additionally, contrasting evidence exists concerning the association between sex and mortality. While some studies indicate significantly higher mortality rates in men [23], others show that the female gender is independently associated with increased mortality in individuals requiring mechanical ventilation [24]. Genetic predisposition may significantly influence vulnerability to blastomycosis, as a study has identified interleukin-6 as a potential susceptibility locus [25]. While certain populations, such as the Hmong community, have been identified as having a higher risk of developing blastomycosis due to specific genetic factors [25], it's important to recognize that genetic susceptibility is not limited to any one ethnic group. The most significant recorded outbreak of blastomycosis occurred in Wisconsin, where 55 individuals contracted the disease over a two-year period, with 40% of the affected population being of Hmong descent [26]. In our report, both patients diagnosed with blastomycosis were White, underscoring that individuals outside of high-risk populations can also be vulnerable. This suggests that other genetic or environmental factors may play a role, and further research is needed to understand the complex interplay between genetics and fungal infection susceptibility across diverse populations.

Obesity poses a significant risk for both community-acquired [13] and healthcare-associated pneumonia [14,15]. Obesity can lead to impaired immune responses, heightened risk of aspiration, reduced lung volumes, and altered ventilation patterns, potentially making individuals more susceptible to lower respiratory tract infections [27]. Furthermore, obesity correlates with chronic diseases like diabetes, heart failure, stroke, gastroesophageal reflux disease, asthma, certain cancers, and MASLD, which also increase the risk of pneumonia [28]. The relationship between obesity and pneumonia is complex, with uncertainties about whether obesity itself or its associated comorbidities predominantly contribute to increased risk. Notably, the "obesity paradox" suggests a protective effect of obesity on pneumonia outcomes [29], as observed in a retrospective study indicating lower mortality rates among obese patients (4% vs. 10% mortality) [30]. However, the mechanisms behind this phenomenon remain unclear and warrant further research.

Obesity, characterized by protracted low-grade inflammation, induces immunosuppression [31]. This condition hampers innate immune responses, affecting monocytes, macrophages, dendritic cells, and natural killer cells, while also impairing cell-mediated immunity [16]. Consequently, susceptibility to fungal infections increases [32]. While leptin, an adipocyte-derived hormone/cytokine that primarily regulates food intake and basal metabolism, is crucial for glucose uptake in effector T cells, chronic systemic leptin secretion in obesity can disrupt CD4+ T cell differentiation and lead to poor T cell responses. Elevated leptin signaling promotes pro-inflammatory T cell subsets, such as Th1 and Th17, contributing to an inflammatory adipose tissue microenvironment [33]. However, it has been shown that IL-17 derived from Th17 cells enhances blastomycosis immunity by recruiting neutrophils. Therefore, more research is needed to understand the complex relationship between hyperleptinemia in obesity and immunity to fungal infections [34]. Furthermore, adipose tissue-derived proinflammatory cytokines and adipocyte-induced T cell activation contribute to T cell population reduction, leading to immunosuppression [35,36]. Additionally, obesity is strongly linked to more severe illness and higher mortality rates in COVID-19 patients [37]. This illustrates further that it is a risk factor not only for bacterial and fungal pneumonia but also for viral pneumonia.

Obesity is frequently linked to vitamin D deficiency [38]. Four main mechanisms are proposed to explain this association: (1) decreased sun exposure, (2) negative feedback from increased 1,25-dihydroxyvitamin D levels in obese individuals reduces 25-hydroxyvitamin D levels, sequestration of vitamin D within adipose tissue, and (4) volumetric dilution [39]. However, the evidence favors sequestration in adipose tissue and volumetric dilution as the primary mechanisms [40,41] for the low vitamin D status commonly observed in overweight and obese individuals.

Diagnosing blastomycosis can be challenging due to nonspecific symptoms and radiographic findings, often necessitating a thorough patient history for accurate diagnosis. Radiographic findings vary and may resemble other pulmonary diseases, emphasizing the importance of clinical suspicion and diagnostic testing [9]. Diagnostic methods encompass microscopic examination, culture-based diagnostics, and non-culture diagnostics such as antibody testing, enzyme immunoassays, and antigen assays [7]. It is important to recognize that while the β-D-glucan test is widely utilized for diagnosing invasive fungal infections such as those caused by *Candida *and *Aspergillus *species [42], it is not commonly used in the evaluation of blastomycosis. This is due to the test's low sensitivity in detecting blastomycosis, attributed to the low levels of 1,3-β-D-glucan in the cell wall of *Blastomyces *[43]. Additionally, elevated triglyceride and bilirubin levels have been demonstrated to interfere with the β-D-glucan assay, potentially leading to an increased rate of false-negative results. As a result, individuals with high triglyceride levels, such as those who are obese or those with hemolyzed samples, may experience reduced test sensitivity. This can complicate the accurate diagnosis of fungal infections in these populations [42]. This likely explains the negative results observed in both of our patients. Antifungal therapy, particularly with itraconazole as first-line treatment, is recommended for all diagnosed cases, with amphotericin B reserved for severe cases [44,45].

Vitamin D serves as a crucial factor in numerous physiological processes, including the modulation of the immune system function by regulating both the innate and adaptive immune response, impacting the production of crucial endogenous antimicrobial peptides, and modulating the inflammatory cascade [46]. Vitamin D plays a crucial role in stimulating the production of cathelicidin, an antimicrobial peptide that directly kills pathogens or binds to their endotoxin, altering their membrane permeability [47]. Additionally, vitamin D regulates the inflammatory cascade by modulating the nuclear factor kappa B pathway [48]. Within the innate immune system, various cell types, such as respiratory epithelial cells, macrophages, monocytes, and dendritic cells, express both the enzyme CYP27B1, necessary for converting inactive 25-hydroxyvitamin D to active 1,25-dihydroxyvitamin D, and the vitamin D receptor (VDR) [49]. Vitamin D activates the T cell VDR, inhibiting Th1-mediated responses and promoting Th2 cell differentiation, along with regulatory T cell development [50]. Given the potent inflammatory effects of TNF and interferon-gamma and the pro-inflammatory profile of Th1 cytokines, optimizing vitamin D status may help regulate inflammation in infected individuals. In contrast, vitamin D deficiency can lead to a dysregulated and more pro-inflammatory state. A study by de Haan et al. [51] showed that vitamin D deficiency (<50 nmol/L) is associated with increased rates of infections, sepsis, 30-day mortality, and in-hospital mortality in critically ill adult patients. Additionally, a link between vitamin D deficiency and the development of blastomycosis has been observed in mammals, specifically dogs, as studies in humans are lacking. A 2021 study demonstrated that dogs with blastomycosis had significantly lower serum 25-hydroxyvitamin D levels at the time of diagnosis compared to healthy controls. Furthermore, baseline serum 25-hydroxyvitamin D levels were significantly lower in dogs that did not survive to hospital discharge, to 30 days post-diagnosis, or to the end-of-study follow-up, compared to those that did, confirming its role in the outcome of blastomycosis [52].

## Conclusions

Clinicians should maintain a heightened awareness of the potential for fungal infections, particularly in obese individuals from endemic areas who do not show improvement with standard treatment for CAP. These patients may present without classic risk factors for fungal infections yet still harbor conditions like vitamin D deficiency, which could compromise their immune response. Therefore, it is recommended to assess vitamin D levels in such individuals, as it may offer valuable insight into their susceptibility to infections.

Moreover, the link between vitamin D deficiency and blastomycosis risk remains an area that warrants further investigation. While current knowledge suggests a possible connection, there is a need for well-designed prospective studies to thoroughly examine this relationship in human populations. Clarifying this association could lead to better preventive and therapeutic strategies for at-risk individuals.

## References

[1] Brown EM, McTaggart LR, Zhang SX, Low DE, Stevens DA, Richardson SE (2013). Phylogenetic analysis reveals a cryptic species Blastomyces gilchristii, sp. nov. within the human pathogenic fungus Blastomyces dermatitidis. PLoS One.

[2] Schwartz IS, Wiederhold NP, Hanson KE, Patterson TF, Sigler L (2019). Blastomyces helicus, a new dimorphic fungus causing fatal pulmonary and systemic disease in humans and animals in Western Canada and the United States. Clin Infect Dis.

[3] O'Dowd TR, Mc Hugh JW, Theel ES, Wengenack NL, O'Horo JC, Enzler MJ, Vergidis P (2021). Diagnostic methods and risk factors for severe disease and mortality in blastomycosis: a retrospective cohort study. J Fungi (Basel).

[4] Smith DJ, Williams SL, Benedict KM, Jackson BR, Toda M (2022). Surveillance for Coccidioidomycosis, Histoplasmosis, and Blastomycosis - United States, 2019. MMWR Surveill Summ.

[5] Ajmal M, Aftab Khan Lodhi F, Nawaz G, Basharat A, Aslam A (2022). Blastomycosis-induced acute respiratory distress syndrome. Cureus.

[6] Furlan AM, Costa Filho FF, Gusfa DW, Tang HM, Avner BS (2024). Blastomycosis complicated by adult respiratory distress syndrome in an immunocompetent adult: a case report and literature review. Cureus.

[7] McBride JA, Gauthier GM, Klein BS (2017). Clinical manifestations and treatment of blastomycosis. Clin Chest Med.

[8] (2014). Feigin and Cherry's textbook of pediatric infectious diseases.

[9] Maini R, Ranjha S, Tandan N (2020). Pulmonary blastomycosis: a case series and review of unique radiological findings. Med Mycol Case Rep.

[10] Rimawi A, Amireh K, Crabtree T, Robinson R (2022). Blastomycosis presenting as a primary tracheal tumor: a rare presentation. Cureus.

[11] Hales CM, Carroll MD, Fryar CD, Ogden CL (2020). Prevalence of obesity and severe obesity among adults: United States, 2017-2018. NCHS Data Brief.

[12] Pi-Sunyer X (2009). The medical risks of obesity. Postgrad Med.

[13] Phung DT, Wang Z, Rutherford S, Huang C, Chu C (2013). Body mass index and risk of pneumonia: a systematic review and meta-analysis. Obes Rev.

[14] Huttunen R, Syrjänen J (2013). Obesity and the risk and outcome of infection. Int J Obes (Lond).

[15] Bochicchio GV, Joshi M, Bochicchio K, Nehman S, Tracy JK, Scalea TM (2006). Impact of obesity in the critically ill trauma patient: a prospective study. J Am Coll Surg.

[16] Andersen CJ, Murphy KE, Fernandez ML (2016). Impact of obesity and metabolic syndrome on immunity. Adv Nutr.

[17] Pereira-Santos M, Costa PR, Assis AM, Santos CA, Santos DB (2015). Obesity and vitamin D deficiency: a systematic review and meta-analysis. Obes Rev.

[18] Ismailova A, White JH (2022). Vitamin D, infections and immunity. Rev Endocr Metab Disord.

[19] Shaikh SR, Beck MA, Alwarawrah Y, MacIver NJ (2024). Emerging mechanisms of obesity-associated immune dysfunction. Nat Rev Endocrinol.

[20] Villacorta Cari E, Leedy N, Ribes JA, Soria J, Myint T (2022). Risk factors of severe blastomycosis and comparison of diagnosis and outcomes between immunocompetent and immunocompromised patients. Mycoses.

[21] McBride JA, Sterkel AK, Matkovic E, Broman AT, Gibbons-Burgener SN, Gauthier GM (2021). Clinical manifestations and outcomes in immunocompetent and immunocompromised patients with blastomycosis. Clin Infect Dis.

[22] Azar MM, Assi R, Relich RF, Schmitt BH, Norris S, Wheat LJ, Hage CA (2015). Blastomycosis in Indiana: clinical and epidemiologic patterns of disease gleaned from a multicenter retrospective study. Chest.

[23] Khuu D, Shafir S, Bristow B, Sorvillo F (2014). Blastomycosis mortality rates, United States, 1990-2010. Emerg Infect Dis.

[24] Rush B, Lother S, Paunovic B, Mooney O, Kumar A (2021). Outcomes with severe blastomycosis and respiratory failure in the United States. Clin Infect Dis.

[25] Merkhofer RM Jr, O'Neill MB, Xiong D (2019). Investigation of genetic susceptibility to blastomycosis reveals interleukin-6 as a potential susceptibility locus. mBio.

[26] Roy M, Benedict K, Deak E (2013). A large community outbreak of blastomycosis in Wisconsin with geographic and ethnic clustering. Clin Infect Dis.

[27] Kornum JB, Nørgaard M, Dethlefsen C (2010). Obesity and risk of subsequent hospitalisation with pneumonia. Eur Respir J.

[28] Formiguera X, Cantón A (2004). Obesity: epidemiology and clinical aspects. Best Pract Res Clin Gastroenterol.

[29] Papagianni M, Tziomalos K (2017). Effects of obesity on the outcome of pneumonia. Expert Rev Endocrinol Metab.

[30] Kahlon S, Eurich DT, Padwal RS, Malhotra A, Minhas-Sandhu JK, Marrie TJ, Majumdar SR (2013). Obesity and outcomes in patients hospitalized with pneumonia. Clin Microbiol Infect.

[31] Muscogiuri G, Pugliese G, Laudisio D, Castellucci B, Barrea L, Savastano S, Colao A (2021). The impact of obesity on immune response to infection: plausible mechanisms and outcomes. Obes Rev.

[32] Lee PP, Lau YL (2017). Cellular and molecular defects underlying invasive fungal infections-revelations from endemic mycoses. Front Immunol.

[33] Green WD, Beck MA (2017). Obesity altered T cell metabolism and the response to infection. Curr Opin Immunol.

[34] McDermott AJ, Klein BS (2018). Helper T-cell responses and pulmonary fungal infections. Immunology.

[35] Al-Mansoori L, Al-Jaber H, Prince MS, Elrayess MA (2022). Role of inflammatory cytokines, growth factors and adipokines in adipogenesis and insulin resistance. Inflammation.

[36] Park HL, Shim SH, Lee EY (2014). Obesity-induced chronic inflammation is associated with the reduced efficacy of influenza vaccine. Hum Vaccin Immunother.

[37] Singh R, Rathore SS, Khan H (2022). Association of obesity with COVID-19 severity and mortality: an updated systemic review, meta-analysis, and meta-regression. Front Endocrinol (Lausanne).

[38] Kaidar-Person O, Person B, Szomstein S, Rosenthal RJ (2008). Nutritional deficiencies in morbidly obese patients: a new form of malnutrition? Part A: vitamins. Obes Surg.

[39] Pourshahidi LK (2015). Vitamin D and obesity: current perspectives and future directions. Proc Nutr Soc.

[40] Moan J, Lagunova Z, Lindberg FA, Porojnicu AC (2009). Seasonal variation of 1,25-dihydroxyvitamin D and its association with body mass index and age. J Steroid Biochem Mol Biol.

[41] Drincic AT, Armas LA, Van Diest EE, Heaney RP (2012). Volumetric dilution, rather than sequestration best explains the low vitamin D status of obesity. Obesity (Silver Spring).

[42] Wright WF, Overman SB, Ribes JA (2011). (1-3)-β-D-glucan assay: a review of its laboratory and clinical application. Lab Med.

[43] Girouard G, Lachance C, Pelletier R (2007). Observations on (1-3)-beta-D-glucan detection as a diagnostic tool in endemic mycosis caused by Histoplasma or Blastomyces. J Med Microbiol.

[44] Chapman SW, Dismukes WE, Proia LA, Bradsher RW, Pappas PG, Threlkeld MG, Kauffman CA (2008). Clinical practice guidelines for the management of blastomycosis: 2008 update by the Infectious Diseases Society of America. Clin Infect Dis.

[45] Limper AH, Knox KS, Sarosi GA (2011). An official American Thoracic Society statement: treatment of fungal infections in adult pulmonary and critical care patients. Am J Respir Crit Care Med.

[46] Gunville CF, Mourani PM, Ginde AA (2013). The role of vitamin D in prevention and treatment of infection. Inflamm Allergy Drug Targets.

[47] Ramanathan B, Davis EG, Ross CR, Blecha F (2002). Cathelicidins: microbicidal activity, mechanisms of action, and roles in innate immunity. Microbes Infect.

[48] Hansdottir S, Monick MM, Lovan N, Powers L, Gerke A, Hunninghake GW (2010). Vitamin D decreases respiratory syncytial virus induction of NF-kappaB-linked chemokines and cytokines in airway epithelium while maintaining the antiviral state. J Immunol.

[49] Mora JR, Iwata M, von Andrian UH (2008). Vitamin effects on the immune system: vitamins A and D take centre stage. Nat Rev Immunol.

[50] Adams JS, Hewison M (2008). Unexpected actions of vitamin D: new perspectives on the regulation of innate and adaptive immunity. Nat Clin Pract Endocrinol Metab.

[51] de Haan K, Groeneveld AB, de Geus HR, Egal M, Struijs A (2014). Vitamin D deficiency as a risk factor for infection, sepsis and mortality in the critically ill: systematic review and meta-analysis. Crit Care.

[52] Jacobs C, Jaffey JA, Trepanier LA, Pritchard JC (2021). Serum 25-hydroxyvitamin D concentrations and mortality in dogs with blastomycosis. Vet J.

